# High Quality of Care Delivery Improves Patient Satisfaction and Quality of Life Outcomes After Breast Augmentation

**DOI:** 10.1093/asj/sjae126

**Published:** 2024-06-14

**Authors:** Kim Phi Luong, Marloes H P ter Stege, Stefan Hummelink, Laura Zaal, Harm P Slijper, Dietmar J O Ulrich

## Abstract

**Background:**

Breast augmentation is one of the most common aesthetic procedures worldwide. Most studies have focused on evaluating the outcome with validated patient-reported outcome measures (PROMs) and factors that may influence them. However, the influence of care delivery, which can be measured with patient-reported experience measures (PREMs), is rarely considered in studies of breast augmentation patients.

**Objectives:**

In this study we aimed to evaluate the associations between PREMs and PROMs in patients who underwent breast augmentation.

**Methods:**

A multicenter cohort study was conducted in breast augmentation patients. Patients completed PREMs, including items such as communication between physician and patient, expectation management, welcome, and hygiene, and the BREAST-Q PROM (satisfaction with breasts and psychosocial, physical, and sexual well-being) preoperatively and 6 months postoperatively. Regression analyses were performed to investigate the associations between PREMs and PROMs.

**Results:**

Overall, 329 patients were included between 2018 and 2022. Univariate regression analysis showed a positive association between PREM and PROM scales. The feeling of being heard (B = −38.39 and B = −18.90), the opportunity to ask questions (B = −9.21), and trust in their physician (B = −39.08) had the highest association with change in the 4 BREAST-Q scales. The multivariable regression analysis showed that the variance in PROMs related to changes in PREMs (19%) was little influenced by patient characteristics (1%).

**Conclusions:**

Patient outcomes are more positive after breast augmentation when patients feel they are being heard, have the opportunity to ask questions, and have trust in their physician. Future studies should be targeted at optimizing patient-reported experience to investigate whether this improves patient-reported outcomes.

**Level of Evidence: 3:**

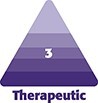

Breast augmentation is one of the most common aesthetic procedures worldwide, with high patient-reported satisfaction postsurgery.^1-5^ Despite the consistent popularity of the procedure over the years and the expanding number of procedures, the surgeon’s perceptions of the treatment outcome can vary from those of the patient.^1^ Because the definition of a successful procedure is mainly determined by the patient, patient-reported outcome measures (PROMs) and patient-reported experience measures (PREMs) are important determinants.

Patient-reported outcomes, including satisfaction and quality of life following breast augmentation, can be measured with the BREAST-Q augmentation module, a validated PROM that is utilized internationally.^6^ The BREAST-Q includes items such as body image (eg, feeling attractive), confidence in social and sexual settings, pain or tightness in the breast area, and breast appearance (eg, symmetry, how the breasts look when clothed or unclothed). The PREM is a reliable questionnaire that provides insight into the patient's experience with the delivered health care.^7^ It includes items such as being taken seriously by the physician, the opportunity to ask all questions, and the clinic's hygiene. PROMs measure the outcomes of a procedure, whereas PREMs measure the impact of the process of care.

To assess whether a procedure or outcome is successful, it is essential to understand which factors have an impact. The majority of patient-reported outcome studies report on investigation of factors such as patient characteristics, technical aspects of surgery, and implant characteristics, yet only a few studies have considered the experiences of patients during their augmentation treatment.^8-12^ There are many aspects of care delivery that potentially influence how patients experience their treatment. For instance, Correia-Sá et al showed that patients who were satisfied with their result defined their preoperative information as more sufficient than did dissatisfied patients.^12^

In other medical fields, studies have shown an association between the process of care delivery and treatment outcomes (PROMs).^13-16^ Aspects such as general treatment information provision, the physician's communication, and trust in the physician have been associated with positive changes in PROMs. In the aesthetic field, PREMs have not been thoroughly investigated for breast augmentation patients. It is important for healthcare centers and providers to understand outcomes from the patient’s perspective, especially in elective surgery, to enhance the quality of care and potentially improve patient-reported outcomes. Therefore, we aimed to assess whether, and to what extent, patient-reported experience measures were associated with patient-reported outcomes as measured by the BREAST-Q at 6 months after breast augmentation.

## METHODS

### Study Design and Setting

This retrospective cohort study included patients who underwent breast augmentation at all 5 locations of the Velthuis Clinics in the Netherlands between July 2018 and April 2022. As part of routine outcome measurement, patients were invited to complete a secure web-based PROM questionnaire (the BREAST-Q) before and after surgery and a PREM questionnaire after their first consultation and their surgery with GemsTracker.^17^ A maximum of 2 reminders were sent.

Patient characteristics were obtained from the electronic health records (age, BMI, current smoking status, history of cosmetic surgery, and education level). Education level was categorized by 3 groups, derived from the International Standard Classification of Education and sorted by level of degree: high (eg, bachelor's degree or equivalent), medium (eg, upper secondary education), and low (eg, primary education).^18^ All patients signed an informed consent for use of their data for research purposes. This study was approved by the local Medical Ethical Review Committee of Radboudumc (2020-6680), following the guidelines of the Declaration of Helsinki.

### Participants

Participants were patients of the age of 18 and older who underwent a primary bilateral breast augmentation. Patients were excluded if they had undergone another breast surgery, abdominoplasty, or liposuction in the abdominal area within 6 months, to obtain a homogeneous group. The exclusion of procedures in the abdominal region was because the BREAST-Q sexual well-being scale addressed questions regarding overall body perception without specific mention of the breasts. Cosmetic procedures in both the breast and abdominal areas were excluded to mitigate potential confounding effects on the study outcome. Patients who had completed the preoperative and postoperative PROMs and PREMs were included in the final analysis.

### Patient-Reported Outcome Measure: BREAST-Q Augmentation Module

Patients completed the BREAST-Q augmentation module, which consisted of 2 domains: patient satisfaction and health-related quality of life.^6,19^ From these domains, 4 scales, including satisfaction with breasts, psychosocial well-being, physical well-being, and sexual well-being, were selected. These scales were independently scored on a 4-point or 5-point Likert scale and converted to a score ranging from 0 to 100 by the Qscore 1.6 software (2009-2022, Memorial Sloan Kettering Cancer Center and the University of British Columbia). A higher score indicated greater satisfaction or quality of life. The BREAST-Q was filled in before surgery and 6 months postoperatively. The change between the preoperative and postoperative PROM scales for each patient was calculated to measure treatment effectiveness with regard to satisfaction and quality of life. The satisfaction with breasts scale was defined as the primary outcome, and the other 3 scales were defined as secondary outcomes.

### Patient-Reported Experience Measures

Patients completed 2 PREMs; the first PREM was sent after their first consultation, and the second PREM was sent 4 months postoperatively. These PREMs were administered to measure the patient's experience with the clinic (eg, accessibility and hygiene) and communication, interpersonal interaction, and information provision between physician and patient. The PREM was a questionnaire commonly employed by hospitals and outpatient care in the Netherlands.^20^ The first PREM consisted of 22 experience questions, and the second PREM comprised 11 experience questions (Appendix, available at www.aestheticsurgeryjournal.com). With the support of the question, “Please choose the five most important factors regarding patient experience that matter to you the most,” 5 questions of each PREM were identified by the patient as most relevant, resulting in a total of 10 items for the current analysis. Both PREMs were scored on a 4- or 5-point Likert scale and had the option of “Not applicable” for a question that might not apply to a patient, or if the patient could not recall their experience.

### Missing Data

On the subscales of the PREMs, 1% of patients indicated that a question did not apply to them or that they could not recall their experience. This percentage was considered missing data completely at random or at random and was imputed with multiple imputations by chained equations (MICE).

### Statistical Analyses

A comparison of baseline patient characteristics and PROM scores between patients who met the inclusion criteria (completers) and those who did not (noncompleters) was performed to assess the potential of selection bias.

A univariate linear regression analysis was performed to assess the relationship between each of the 10 PREM items and the 4 PROM change scores. The effect size of each PREM item was determined with beta coefficients. An effect size larger than or equal to 8 for satisfaction with breasts, 10 for psychosocial well-being, 7 for physical well-being, and 10 for sexual well-being was regarded as clinically relevant.^21^

All 10 PREM items were entered simultaneously in the same model and the explained variance (R2) was calculated, to determine to what extent the experience with healthcare delivery could explain the variation in patient-reported satisfaction and quality of life between patients. Another multivariable regression model was conducted to adjust for potential variance in patient characteristics, including age, BMI, smoking status, history of cosmetic surgery, and education level.

All data were conducted with R version 3.6.3 (R Foundation for Statistical Computing, Vienna, Austria). A *P* value of <.05 was considered statistically significant.

## RESULTS

### Descriptives

**Figure 1.**
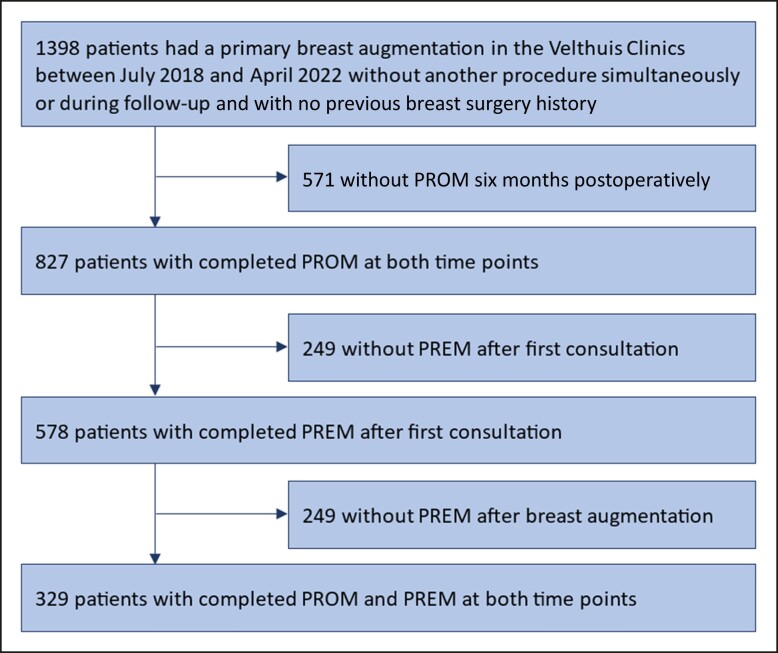
Flowchart of patient inclusion.

A total of 1398 female patients underwent a primary breast augmentation between July 2018 and April 2022, of which 329 patients were included for analysis (Figure 1). Patients were between 18 and 58 years old, with a mean age of 29.8 years. Table 1 shows the patient characteristics and baseline patient-reported outcomes. There were no significant differences in patient characteristics or baseline patient-reported outcomes between completers and noncompleters. The change in preoperative and postoperative PROM scores was significant for all 4 scales. It exceeded the minimal important difference (MID), resulting in a clinically relevant difference (Table 2). These outcomes have been reported in a previous descriptive cohort study.^5^

**Table 1.** Baseline Patient Characteristics of Included vs Not Included Patients

	Included 329	Not included 1069	*P* value	Effect sizes
Age (years)^a^	29.8 ± 8.7	30.6 ± 8.9	.13	0.10
BMI (kg/m^2^)^a^	21.7 ± 3.3	21.9 ± 3.8	.63	0.03
Smoking status (yes)	67 (20.4)	248 (23.2)	.32	0.03
Cosmetic surgery in the past (yes)	28 (8.5)	112 (10.5)	.35	0.02
Education level				
Low	9 (2.7)			
Medium	153 (46.5)			
High	167 (50.8)			
BREAST-Q scores^a^				
Satisfaction with breasts	20.9 ± 14.6	22.6 ± 14.7	.06	0.12
Psychosocial well-being	42.1 ± 17.2	42.9 ± 17.7	.50	0.04
Physical well-being	95.4 ± 8.6	94.8 ± 9.5	.28	0.28
Sexual well-being	33.2 ± 16.0	35.0 ± 16.2	.08	0.11

^a^Mean ± standard deviation. BMI, body mass index.

**Table 2.** Outcome Measurements at Baseline and 6 Months Postoperatively

*n* = 329	Preoperative	Postoperative	Δ	MID
BREAST-Q scores^a^				
Satisfaction with breasts	20.9 ± 14.6	79.1 ± 14.7^b^	58.2 ± 19.5	8
Psychosocial well-being	42.1 ± 17.2	77.5 ± 17.0^b^	35.4 ± 22.6	10
Physical well-being	95.4 ± 8.6	76.2 ± 19.2^b^	19.2 ± 19.3	7
Sexual well-being	33.2 ± 16.0	80.3 ± 14.8^b^	47.2 ± 20.0	10

^a^Median ± standard deviation. ^b^*P* value <.001. MID, minimal important difference.

Table 3 presents the distribution of the PREM answers of the top 10 most important PREM items chosen by patients, along with missing data, by question. Patients had high PREM item scores, varying from 83.6% to 97.9% after their first consultation and 64.7 to 93.3% after surgery.

**Table 3.** Distribution of Patient-Reported Experience Measure Answers per Question (*n* = 329)

PREMs items					
After consultation	Welcome	Serious	Patient-physician time	Expert	Questions
Always	288 (87.5)	312 (94.8)	275 (83.6)	322 (97.9)	295 (89.7)
Often	37 (11.3)	16 (4.9)	43 (13.1)	7 (2.1)	30 (9.1)
Sometimes	4 (1.2)	1 (0.3)	10 (3.0)	0 (0.0)	4 (1.2)
Never	0 (0.0)	0 (0.0)	1 (0.3)	0 (0.0)	0 (0.0)
After treatment	Expectations	Pros & cons	Listening	Trust	Hygiene
Always	246 (74.8)	213 (64.7)	265 (80.6)	293 (89.1)	307 (93.3)
Often	70 (21.3)	92 (28.0)	51 (15.5)	26 (7.9)	9 (2.7)
Sometimes	8 (2.4)	12 (3.7)	9 (2.7)	5 (1.5)	0 (0.0)
Rarely	4 (1.2)	6 (1.8)	0 (0.0)	1 (0.3)	0 (0.0)
Never	0 (0.0)	0 (0.0)	1 (0.3)	1 (0.3)	0 (0.0)
NA	1 (0.3)	1 (0.3)	3 (0.9)	3 (0.9)	13 (4.0)

Values in parentheses are percentages. Welcome = Did you feel welcome at the clinic? Serious = Did the physician take you seriously? Patient-physician time = Did the physician have enough time for you? Expert = Was the physician knowledgeable? Questions = Did you get the opportunity to ask all your questions of the physician? Expectations = Did you receive information in advance about care in the clinic, so you knew what to expect? Pros & cons = Were the advantages and disadvantages of the treatment or surgery explained to you? Listening = Did the healthcare providers listen to you well? Trust = Do you have confidence in the expertise of the healthcare providers? Hygiene = Do the clinic staff work in a clean manner? NA, not applicable, or patient cannot recall their experience.

### Association of Patient-Reported Experience Measures With Patient-Reported Outcome Measures

**Figure 2.**
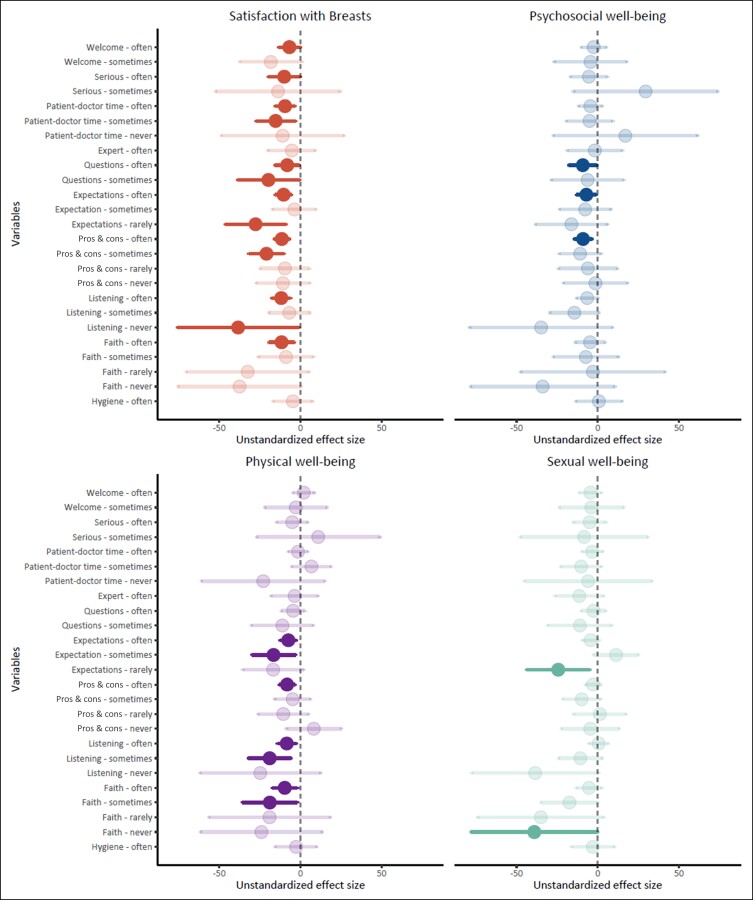
Forest plot of the regression coefficients of the 10 aspects of the PREMs for each subscale of the BREAST-Q: satisfaction with breasts (red), psychosocial well-being (blue), physical well-being: chest (purple), and sexual well-being (green). The regression coefficients are presented as dots and 95% confidence intervals as horizontal lines. These coefficients were compared to the reference outcome “always,” which meant 100% satisfaction with the PREM. The dashed vertical line represents the “no effect” line. Significant variables are depicted with a higher opacity than nonsignificant variables. All significant variables can be seen on the left side of the no effect line, meaning patients who were less satisfied compared to patients who were always satisfied with the PREM experience had a lower treatment effect on the BREAST-Q scales. The most influential factors associated with satisfaction with breasts, psychosocial well-being, physical well-being, and sexual well-being were listening, questions, listening, and trust, respectively.

The univariate regression analysis showed a positive association between PREM and PROM scales, with “listening” (B = −38.39 [−0.94, −75.83]) on the PREM having the highest association with the satisfaction with breasts change score on the PROM; “questions” (B = −9.21 [−0.73, −17.68]) with psychosocial well-being; “listening,” again, (B = −18.90 [−5.92, −31.87]) with physical well-being; and “trust” (B = −39.08 [−0.17, −77.99]) with sexual well-being (Figure 2, and Supplemental Table 1 available at www.aestheticsurgeryjournal.com). For instance, if a patient reported feeling “never” heard by the physician, and another patient reported feeling “always” heard, the change score for satisfaction with breasts at 6 months postoperatively decreased by 38.39 points on a scale of 0 to 100. Similarly, if a patient felt “often” given the opportunity to ask questions compared to another patient who felt “always” given that opportunity, the change score in psychosocial well-being at 6 months postoperatively decreased by 9.21 points on a scale of 0 to 100.

Multivariable regression analysis showed that, when combining all the individual PREM items in 4 models for the different PROM scales, the PREMs explained 8% to 18% of the variation in patient-reported outcomes between patients who underwent breast augmentation (Supplemental Table 1). The PREM had the strongest association with the total change score in satisfaction with breasts on the PROM scales, with 18% of the variance explained by the PREM. When adjusted for patient characteristics, 1% was added to the explained variance of 19% for the satisfaction with breasts scale, 3% of 11% for psychosocial well-being, 1% of 17% for physical well-being, and 3% of 11% for sexual well-being (Supplemental Table 1).

## DISCUSSION

In this study we aimed to assess whether patient-reported experience measures are associated with patient-reported outcomes 6 months after breast augmentation. When considering the 4 outcomes of the BREAST-Q, the largest effects were found for the following PREM items: if the patient felt that the physician was listening to them most affected satisfaction with breasts; if the patient felt they had the opportunity to ask questions most affected psychosocial well-being; if the patient felt that the physician was listening to them had the largest effect on physical well-being; and if the patient trusted their physician most affected sexual well-being. Combining all PREM items, the explained variance of the BREAST-Q change score established a percentage of 8 to 18. An additional 1% to 3% was explained when adjusting for patient characteristics, indicating that patient characteristics were less significant than PREM items. Overall, it was observed that better healthcare experiences were associated with greater patient-reported treatment satisfaction and several aspects of well-being.

The findings regarding patient-reported outcomes were in line with previously reported studies that described higher satisfaction and quality of life postoperatively.^2-4,8^ The association between the 4 BREAST-Q outcome scales and items on the PREM has not been previously reported in breast augmentation patients, but findings were similar to those of comparable studies with different patient populations for the 3 items of the PREM (listening, questions, and trust).^13-16^

In the present study, the 2 outcomes on BREAST-Q satisfaction with breasts and physical well-being had the strongest association with listening. In previous studies this item was divided under “physician’s communication and competence,” and all these studies reported a high association with patient-reported experience.^14-16^ The BREAST-Q outcome psychosocial well-being was most strongly associated with questions. This aspect may also be considered part of the “physician's communication and competence” because strong communication may lead to a better understanding of the importance of providing patients with opportunities to ask questions. Communication in general has been mentioned in many studies as an important factor in expectation management. Providing adequate and tailored information for the individual patient may lead to altered expectations.^22^ It is known that patients with high or unrealistic expectations are more dissatisfied.^23^ In addition, patient satisfaction with preoperative information and the interaction between patient and physician have been observed to influence satisfaction with breasts and the overall outcome.^24^ Therefore, it would not be surprising that it also affects psychosocial well-being.

In our study we found that “trust in their physician” had the highest association with the BREAST-Q outcome for sexual well-being. This PREM item was also observed as one of the most important by physicians and nurses in the study of Black et al for a patient population undergoing knee or hip replacement, even though these patients completed a different PROM.^13^ Additionally, they found a strong association with “sufficient explanation and involvement”; this aspect may be comparable to “general information” and “treatment information,” which were the strongest items associated with outcomes in 3 other studies.^14-16^ These aspects can be compared to PREM information items regarding “expectation management” and the “advantages and disadvantages” of the procedure in the current study. The first item was also associated with all 4 BREAST-Q outcomes, and the second associated with all but sexual well-being. However, these 2 items were less strongly associated than listening, questions, and trust. One of the reasons may be that patients believe it is more important or place more value on feeling comfortable during their consultation and treatment than receiving adequate information regarding their procedure. Birkhäuer et al conducted a meta-analysis regarding health outcomes and trust in healthcare professionals.^25^ They observed that patients reported higher quality of life scores if they trusted their healthcare professional more. Further studies should focus on the approach to trust in the clinical setting.

The strengths of this study included the large sample of patients, the internationally recognized and validated PROMs, and the patient-level data. However, it is essential to acknowledge various limitations of this study. First, within this study ceiling effects (>90% best answer) were observed for the items “serious,” “expert,” and “hygiene” on the PREM questionnaire. Patients scored very high on the PREM questionnaire, with almost all PREM items scoring a 4 or 5, respectively, for the 4-point or 5-point Likert scale questions. This ceiling effect may result in less variability, leading to a weaker association with the PROMs, but the PREM utilized was a commonly administered questionnaire in the Netherlands and required in the daily practice of outpatient clinics. Currently, a new version of this PREM has been developed and the scoring system has been adjusted to a 10-point VAS score. In future studies this PREM may overcome this observed ceiling effect.

Second, among the cohort of 1398 patients undergoing primary breast augmentation, only 329 patients were included, relying on questionnaire compliance. Nonetheless, the inclusion of all 1398 patients would not be anticipated to yield divergent results, because no significant differences were observed in patient characteristics and baseline patient-reported outcomes measured with BREAST-Q between the included (*n* = 329) and excluded patients (*n* = 1069). Moreover, we believe that the compliance accurately reflects daily clinical practice, ensuring the relevance and generalizability of our results.

The clinical relevance of the findings of this study is clear: improving patient experiences can result in greater satisfaction and quality of life for patients undergoing breast augmentation. It should be noted that the present study did not focus on causality, and that it is possible that a better patient-reported outcome resulted in a better patient-reported experience. Future studies and clinical implementation should focus on how to improve patients’ experiences, with specific attention to the physician's communication and competence. Moreover, longitudinal studies would be valuable to assess the long-term impacts of PREMs on PROMs. Patients have a strong need to be listened to and ask questions. Having trust in the physician is another aspect that should be noted. However, to improve these experiences, the causality of patient-reported experiences and patient-reported outcomes should be investigated to establish a foundation for how changes in healthcare should be approached. It is recommended that healthcare management and physicians establish an environment conducive to open communication, with regard to setting and emotional well-being, in which patients are provided with adequate time to engage in questioning and discuss their concerns. Physicians should focus on building a bond with their patients and provide sufficient time for patients to express themselves and leave room for questions, although this is challenging with the ever-increasing time pressure in outpatient clinics. Conducting a qualitative study to determine how physicians can overcome these challenges by interviewing patients after their initial consultation regarding their experiences may be of added value.

## CONCLUSIONS

Breast augmentation patients will be more satisfied when they feel they are being heard, have the opportunity to ask questions, and have trust in their physician. Future studies regarding optimization of the patient-reported experience would be of additional value in improving patient-reported outcomes in breast augmentation patients.

## Supplemental Material

This article contains supplemental material located online at www.aestheticsurgeryjournal.com.

## Supplementary Material

sjae126_Supplementary_Data
