# Supplementary material for: High Quality of Care Delivery Improves Patient Satisfaction and Quality of Life Outcomes After Breast Augmentation
Source: Aesthet Surg J. 2024 Jun 14;44(10):NP686–94. doi: 10.1093/asj/sjae126 (PMC11403808; doi:10.1093/asj/sjae126)
Supplement: sjae126_Supplementary_Data [file sjae126_supplementary_data.zip › Appendix,.docx]

**Appendix.** PREM Questionnaire after Consultation Translated from Dutch to English*

| **PREM after consultation** | | | | | |
| --- | --- | --- | --- | --- | --- |
|  | **4-point Likert scale^θ^** | | | | |
| **Accessibility** |  |  |  |  |  |
| Was it a problem to reach the clinic by phone during the day? | 1 | 2 | 3 | 4 | NA |
| Were you able to visit the clinic at a time that was convenient for you? | 1 | 2 | 3 | 4 | NA |
| Was the clinic easy to find? | 1 | 2 | 3 | 4 |  |
| **Reception at the clinic** | | | | | |
| Did you feel welcome at the clinic? ^†^ | 1 | 2 | 3 | 4 |  |
| Was the receptionist helpful? | 1 | 2 | 3 | 4 |  |
| Did the receptionist give you personal attention? | 1 | 2 | 3 | 4 |  |
| **Facility and waiting time of the clinic** | | | | | |
| Were you assisted within 15 minutes of the scheduled time? | 1 | 2 | 3 | 4 |  |
| Were there sufficient amenities in the clinic’s waiting area (such as magazines, refreshments, toys, brochures)? | 1 | 2 | 3 | 4 |  |
| Was the clinic clean? | 1 | 2 | 3 | 4 |  |
| Did the clinic provide sufficient privacy (for example, at the reception, while changing, during conversations, handling information confidentially?) | 1 | 2 | 3 | 4 |  |
| **Interaction with the physician** | | | | | |
| Did the physician take you seriously? ^†^ | 1 | 2 | 3 | 4 |  |
| Did the physician listen attentively to you? | 1 | 2 | 3 | 4 |  |
| Did the physician have enough time for you? ^†^ | 1 | 2 | 3 | 4 |  |
| Was the physician knowledgeable? ^†^ | 1 | 2 | 3 | 4 |  |
| **Information provided by the physician** | | | | | |
| Did the physician inform you in advance why the chosen treatment was applicable to you? | 1 | 2 | 3 | 4 |  |
| Did the physician inform you well about the treatment? | 1 | 2 | 3 | 4 |  |
| Did the physician inform you about the expected outcome of the treatment? | 1 | 2 | 3 | 4 |  |
| Did the physician inform you in an understandable way about the pros and cons of the treatment? | 1 | 2 | 3 | 4 |  |
| **Communication by the physician** | | | | | |
| Did the physician explain things to you in an understandable way? | 1 | 2 | 3 | 4 |  |
| Did you get the opportunity to ask all your questions to the physician? ^†^ | 1 | 2 | 3 | 4 |  |
| Was the information you received from the physician tailored to your personal situation? | 1 | 2 | 3 | 4 |  |
| Were you able to participate in the decision-making about the treatment? | 1 | 2 | 3 | 4 |  |

PREM, patient-reported outcome measure; NA, not applicable.

*Questions translated from the original Dutch questionnaire. This English questionnaire is not validated and is used to illustrate the Dutch PREM.

^θ^ Answer options for the 4-point Likert scale were “never, rarely, often, always”. To illustrate the PREM, numbers are shown instead of the answer options.

† The five most important factors chosen by the patients: welcome, serious, patient-physician time, expert and questions.

PREM Questionnaire after treatment Translated from Dutch to English*

| **PREM after treatment** | | | | | | | |
| --- | --- | --- | --- | --- | --- | --- | --- |
|  | **5-point Likert scale^θ^** | | | | | | |
| **Experiences during your treatment:** |  |  |  | |  |  |  |
| Did you receive information in advance about the care in the clinic, so you knew what to expect? ^†^ | 1 | 2 | 3 | 4 | | 5 | NA |
| Did you decide together with the healthcare providers which care or treatment you would receive? | 1 | 2 | 3 | 4 | | 5 | NA |
| Were the advantages and disadvantages of the treatment or surgery explained to you? ^†^ | 1 | 2 | 3 | 4 | | 5 | NA |
| Did you receive clear information about how your medications work? | 1 | 2 | 3 | 4 | | 5 | NA |
| Did you receive advice from healthcare providers on how to manage your illness or symptoms in a home setting? | 1 | 2 | 3 | 4 | | 5 | NA |
| Did the healthcare providers listen to you well? ^†^ | 1 | 2 | 3 | 4 | | 5 | NA |
| Do you have confidence in the expertise of the healthcare providers? ^†^ | 1 | 2 | 3 | 4 | | 5 | NA |
| Is there a good collaboration among the healthcare providers in the clinic? | 1 | 2 | 3 | 4 | | 5 | NA |
| Do the various appointments align well with each other? | 1 | 2 | 3 | 4 | | 5 | NA |
| Do clinic staff pay attention to safety? (Which you might notice in their way of working and their focus on medication safety, for example) | 1 | 2 | 3 | 4 | | 5 | NA |
| Do the clinic staff work cleanly?^†^ | 1 | 2 | 3 | 4 | | 5 | NA |

PREM, patient-reported outcome measure; NA, not applicable or patient cannot recall

*Questions translated from the original Dutch questionnaire. This English questionnaire is not validated and is used to illustrate the Dutch PREM.

^θ^ Answer options for the 4-point Likert scale were “never, rarely, sometimes, often, always”. To illustrate the PREM, numbers are shown instead of the answer options.

† The five most important factors chosen by the patients: expectations, pros and cons, listening, trust and hygiene.
