# Supplementary material for: High Quality of Care Delivery Improves Patient Satisfaction and Quality of Life Outcomes After Breast Augmentation
Source: Aesthet Surg J. 2024 Jun 14;44(10):NP686–94. doi: 10.1093/asj/sjae126 (PMC11403808; doi:10.1093/asj/sjae126)
Supplement: sjae126_Supplementary_Data [file sjae126_supplementary_data.zip › Supplemental_Table_1 (3).docx]

**Supplemental Table 1.** Unadjusted beta-coefficients of change score in PROM subscale with a greater PREM-score^1^

|  | **Change score in PROM subscales (95% CI)** | | | |
| --- | --- | --- | --- | --- |
| **PREM – difference of 100% satisfaction compared to:** | **Satisfaction with Breasts** | **Psychosocial well-being** | **Physical well-being: chest** | **Sexual well-being** |
| *Patient experience after first consultation* |  |  |  |  |
| Welcome  Always  Often  Sometimes | [reference]  -6.83 [-13.48; -0.18]*  -18.21 [-37.38; 0.96] | [reference]  -2.53 [-10.31; 5.25]  -4.44 [-26.87; 17.98] | [reference]  2.02 [-4.62; 8.65]  -2.88 [-22.02; 16.25] | [reference]  -4.52 [-11.39; 2.35]  -3.96 [-23.77; 15.84] |
| Serious  Always  Often  Sometimes | [reference]  -10.00 [-19.80; -0.20]*  -13.75 [-52.05; 24.55] | [reference]  -5.47 [-16.85; 5.91]  29.47 [-15.00; 73.93] | [reference]  -5.10 [-14.83; 4.63]  10.96 [-27.05; 48.97] | [reference]  -4.99 [-15.08; 5.11]  -8.42 [-47.87; 31.02] |
| Patient-physician time  Always  Often  Sometimes  Never | [reference]  -9.50 [-15.68; -3.31]*  -15.26 [-27.41; -3.11]*  -10.96 [-48.76; 26.84] | [reference]  -4.54 [-11.83; 2.75]  -5.05 [-19.37; 9.27]  16.94 [-27.60; 61.50] | [reference]  -1.52 [-7.73; 4.70]  6.71 [-5.50; 18.92]  -22.89 [-60.88; 15.11] | [reference]  -3.39 [-9.84; 3.05]  -10.23 [-22.88; 2.42]  -5.93 [-45.30; 33.44] |
| Expert  Always  Often | [reference]  -5.34 [-20.01; 9.34] | [reference]  -1.82 [-18.83; 15.18] | [reference]  -3.61 [-18.12; 10.89] | [reference]  -11.40 [-26.39; 3.60] |
| Questions  Always  Often  Sometimes | [reference]  -8.17 [-15.46; -0.89]*  -19.71 [-38.84; -0.58]* | [reference]  -9.21 [-17.68; -0.73]*  -6.27 [-28.55; 16.00] | [reference]  -4.58 [-11.83; 2.68]  -11.13 [-30.20; 7.94] | [reference]  -2.67 [-10.21; 4.88]  -11.03 [-30.84; 8.78] |
| *Patient experience after treatment* |  |  |  |  |
| Expectations  Always  Often  Sometimes  Rarely | [reference]  -10.39 [-15.41; -5.37]*  -3.76 [-17.14; 9.62]  -27.63 [-46.40; -8.87]* | [reference]  -7.01 [-12.98; -1.05]*  -7.75 [-23.60; 8.10]  -16.25 [-38.49; 5.99] | [reference]  -7.47 [-12.51; -2.44]*  -16.54 [-29.90; -3.18]*  -16.79 [-35.54; 1.96] | [reference]  -4.35 [-9.59; 0.89]  11.26 [-2.69; 25.21]  -24.36 [-43.94; -4.79]* |
| Pros & cons  Always  Often  Sometimes  Rarely  Never | [reference]  -11.48 [-16.05; -6.90]*  -20.89 [-31.77; -10.02]*  -9.56 [-24.73; 5.62]  -10.76 [-27.34; 5.83] | [reference]  -9.08 [-14.54; -3.62]*  -10.86 [-23.88; 2.16]  -6.11 [-24.28; 12.05]  -1.45 [-21.30; 18.41] | [reference]  -8.24 [-12.89; -3.60]*  -4.81 [-15.87; 6.26]  -10.56 [-26.00; 4.88]  8.21 [-8.66; 25.08] | [reference]  -2.91 [-7.82; 1.99]  -9.88 [-21.55; 1.79]  1.28 [-15.00; 17.56]  -4.58 [-22.38; 13.21] |
| Listening  Always  Often  Sometimes  Never | [reference]  -11.82 [-17.51; -6.13]*  -6.88 [-19.50; 5.73]  -38.39 [-75.83; -0.94]* | [reference]  -6.49 [-13.22; 0.24]  -14.40 [-29.58; 0.78]  -34.88 [-79.00; 9.23] | [reference]  -8.43 [-14.10; -2.75]*  -18.90 [-31.87; -5.92]*  -24.75 [-61.90; 12.41] | [reference]  0.54 [-5.43; 6.51]  -10.76 [-24.24; 2.72]  -38.49 [-77.68; 0.71] |
| Trust  Always  Often  Sometimes  Rarely  Never | [reference]  -11.62 [-19.29; -3.95]*  -8.90 [-25.91; 8.10]  -32.50 [-70.27; 5.26]  -37.50 [-75.27; 0.26] | [reference]  -4.72 [-13.84; 4.39]  -7.36 [-27.41; 12.69]  -2.96 [-47.49; 41.57]  -33.96 [-78.49; 10.57] | [reference]  -9.68 [-17.39; -1.97]*  -18.82 [-35.69; -1.95]*  -19.02 [-56;48; 18.45]  -24.02 [-61.48; 13.45] | [reference]  -5.41 [-13.38; 2.57]  -17.48 [-35.00; 0.04]  -35.08 [-73.99; 3.83]  -39.08 [-77.99; -0.17]* |
| Hygiene  Always  Often | [reference]  -4.70 [-16.74; 7.35] | [reference]  0.67 [-13.59; 14.93] | [reference]  -2.72 [-15.63; 10.19] | [reference]  -3.03 [-16.27; 10.22] |
| R2, Explained variance (%)  Adjusted R2 | 18%  11% | 8%  1% | 16%  9% | 8%  1% |
| *Adjusted with patient characteristics*  R2, Explained variance (%)  Adjusted R2 (%) | 19%  11% | 11%  2% | 17%  9% | 11%  2% |

^1^Univariate regression analysis of the association between the ten aspects of experience with delivered healthcare (PREM) and satisfaction and well-being outcome after surgery (PROM, BREAST-Q Augmentation module scales), shown as beta-coefficients with 95% confidence intervals. The bottom row (R2, adjusted R2 and ‘Adjusted with patient characteristics’ R2, adjusted R2) presents the results of the multivariable regression analysis and adjusted multivariable regression analysis for patient characteristics. The numbers show how much of the variation in the subscales of the PROMs is explained by the PREM, and how much of the variation in the subscales of the PROMs is explained by the PREM adjusted for patient characteristics including age, BMI, smoking status, cosmetic surgery in the past and educational level.

* P<0.05

If a patient reported feeling ‘never’ heard by the physician, while another patient reported feeling ‘always’ heard, the change score in ‘Satisfaction with Breasts’ at six months postoperatively would decrease by 38.39 points on a scale of 0 to 100.

Similarly, if a patient felt ‘often’ given the opportunity to asks question compared to another patient who felt ‘always’ given that opportunity, the change score in ‘Psychosocial well-being’ at six months postoperatively would decrease by 9.21 points on a scale of 0 to 100.
